# Muscle metabolic remodelling patterns in Duchenne muscular dystrophy revealed by ultra-high-resolution mass spectrometry imaging

**DOI:** 10.1038/s41598-021-81090-1

**Published:** 2021-01-21

**Authors:** Ivana Dabaj, Justine Ferey, Florent Marguet, Vianney Gilard, Carole Basset, Youssef Bahri, Anne-Claire Brehin, Catherine Vanhulle, France Leturcq, Stéphane Marret, Annie Laquerrière, Isabelle Schmitz-Afonso, Carlos Afonso, Soumeya Bekri, Abdellah Tebani

**Affiliations:** 1 Department of Neonatal Pediatrics, Intensive Care and Neuropediatrics, Rouen University Hospital, 76031 Rouen, France; 2 Normandie Univ, UNIROUEN, CHU Rouen, INSERM U1245, 76000 Rouen, France; 3 Department of Metabolic Biochemistry, Rouen University Hospital, 76031 Rouen, Cedex France; 4 Department of Pathology, Rouen University Hospital, Rouen, France; 5 Department of Neurosurgery, Rouen University Hospital, Rouen, France; 6 Normandie Univ, COBRA UMR 6014 Et FR 3038 Univ Rouen; INSA Rouen; CNRS IRCOF, 1 Rue TesnieÌre, 76821 Mont-Saint-Aignan Cedex, France; 7 Department of Genetics and Reference Center for Developmental Disorders, Normandy Center for Genomic and Personalized Medicine, Normandie Univ, UNIROUEN, Inserm U1245 and Rouen University Hospital, 76000 Rouen, France; 8 APHP, Laboratoire de Génétique Et Biologie Moléculaire, HUPC Cochin, Paris, France

**Keywords:** Neuromuscular disease, Metabolomics, Mass spectrometry

## Abstract

Duchenne muscular dystrophy (DMD) is a common and severe X-linked myopathy, characterized by muscle degeneration due to altered or absent dystrophin. DMD has no effective cure, and the underlying molecular mechanisms remain incompletely understood. The aim of this study is to investigate the metabolic changes in DMD using mass spectrometry-based imaging. Nine human muscle biopsies from DMD patients and nine muscle biopsies from control individuals were subjected to untargeted MSI using matrix-assisted laser desorption/ionization Fourier-transform ion cyclotron resonance mass spectrometry. Both univariate and pattern recognition techniques have been used for data analysis. This study revealed significant changes in 34 keys metabolites. Seven metabolites were decreased in the Duchenne biopsies compared to control biopsies including adenosine triphosphate, and glycerophosphocholine. The other 27 metabolites were increased in the Duchenne biopsies, including sphingomyelin, phosphatidylcholines, phosphatidic acids and phosphatidylserines. Most of these dysregulated metabolites are tightly related to energy and phospholipid metabolism. This study revealed a deep metabolic remodelling in phospholipids and energy metabolism in DMD. This systems-based approach enabled exploring the metabolism in DMD in an unprecedented holistic and unbiased manner with hypothesis-free strategies.

## Introduction

Duchenne muscular dystrophy (DMD) is the most common muscular dystrophy in children, with an incidence of 1 in 5000 live-born boys annually and an estimated prevalence of 15.9 per 100,000 boys in the USA and 19.5 per 100,000 boys in the UK^1–3^. This lethal X-linked recessive neuromuscular disorder is caused by mutations in the *DMD* gene resulting in absent or reduced functional dystrophin (DYS). As in many genetic diseases, DMD exhibits a continuum of disease severity, which may be correlated to the presence or absence of a functional protein. Becker muscular dystrophy is a milder form of dystrophinopathy in which DYS levels are reduced, but a residual partially functional protein remains^3^. The dystrophin-associated protein complex underlies the link between the extracellular matrix and the cytoskeleton, thus ensuring the skeletal muscle strength. Dystrophin dysfunction results in severe architectural muscle changes with subsequent degeneration and impaired regeneration^4,5^. Dystrophin has been shown to be temporo-spatially expressed by discrete neuronal populations, mainly in the pyramidal cells of the hippocampus, in the amygdala, in Purkinje cells and granular neurons of the cerebellum. Thus, DYS is involved in several cognitive functions and human brain development, although the precise mechanisms remain unclear^6^. Muscle weakness and motor delay are usually early symptoms. In the absence of steroid treatment, the ambulation loss occurs at about 10 years of age and near paralysis by age 20, with scoliosis, loss of upper limb function, cardiac involvement, and respiratory insufficiency. Brain dysfunction also occurs at the start of various neurobehavioral developmental disorders, such as intellectual disability, or more specific cognitive disorders, delayed speech and coordination acquisition, executive dysfunction, attention deficit disorder and autism spectrum disorders^1–3,7^. To date, there is no cure for DMD, but the life expectancy of DMD patients has improved owing to steroid treatments and better care. DMD patients lose the ability to walk around 13–14 years of age and eventually die in their 30 s due to cardiopulmonary complications^1,3^. However, numerous clinical trials and experimental approaches are ongoing, such as exon skipping, gene therapy, myostatin inhibitors, utrophin modulation, CRISPR/Cas9 suppression of stop codons and stem cell therapy^8^. Validated outcome measures are required to assess the potential benefits of these treatments. Currently, several functional tests are used such as the 6 min walk test, the North Star ambulatory assessment, and the performance of upper limb test or time items in addition to muscular imaging. However, these tests are often ineffective, and their results do not necessarily correlate with the patient’s condition^9^. Owing to wide phenotypic variability, reliable outcome measurements that would enable assessing treatment efficacy remain tentative, and new biomarkers must be identified to better understand the DMD pathophysiology and more accurately evaluate patient prognosis and follow-up^1^. Metabolic impairments have been associated with DMD since the 1970s^10–13^. However, the advent of new omics technologies allows exploring the metabolism and its components in an unprecedented holistic and unbiased manner with hypothesis-free strategies^14^. Metabolites are small organic molecules involved in enzymatic reactions to form the metabolism. The “metabolome” refers to all metabolites present in a given biological system, fluid, cell, or tissue. Metabolomics is the omics technology that enables exploring the metabolome by comprehensively measuring metabolite levels in a given biological sample^15^. Its main goal is to parse the biochemical changes in a biological system by probing the metabolite variations related to genetic, environmental, drug, dietary and other factors or interventions. Mass spectrometry is a widely used metabolomics technology because of its high detection sensitivity, metabolome coverage and rapid data acquisition turnover^16^. The spatial distribution of metabolites can be tracked holistically and systematically, which is highly valuable for gaining a mechanistic understanding of biological processes. Mass spectrometry imaging (MSI) achieves this by simultaneously revealing the spatial distribution of multiple molecules in a single experiment from various biological samples, particularly tissue Sects.^17^. Thus, this technology shows promising translational potential in the biomedical field^18,19^. MSI has been applied to study DMD muscle biopsies using matrix-assisted laser desorption/ionization time-of-flight (MALDI-TOF) imaging and TOF secondary ion mass spectrometry. Previous investigations highlighted lipid compositional changes^20,21^ or Krebs cycle intermediates^22^ in muscles from DMD mouse models and DMD patients^23^. However, the relatively low mass resolution of the TOF–MS instruments makes peak annotation very challenging. Thus, powerful new mass spectrometry instruments based on Fourier-transform ion cyclotron resonance (FTICR) offer better analytical performance in terms of resolution, sensitivity and specificity^24–26^. Thus, this technology offers unprecedented exhaustive metabolome coverage and non-ambiguous molecular formula assignments^26^.

This work presents the first study ever published using MSI based on MALDI coupled with FTICR to explore muscle metabolic remodelling in DMD patients. Given the scope of the study and the high analytical superiority of the platform, this investigation allowed holistically parsing the complex metabolic remodelling in DMD tissues, revealing the DMD-associated molecular metabolic impairments and deregulations, aiming to open new avenues for deeper biological investigations to uncover new therapeutic targets, biomarkers and diagnostic tools.

## Results

### Histological findings

**Figure 1:**
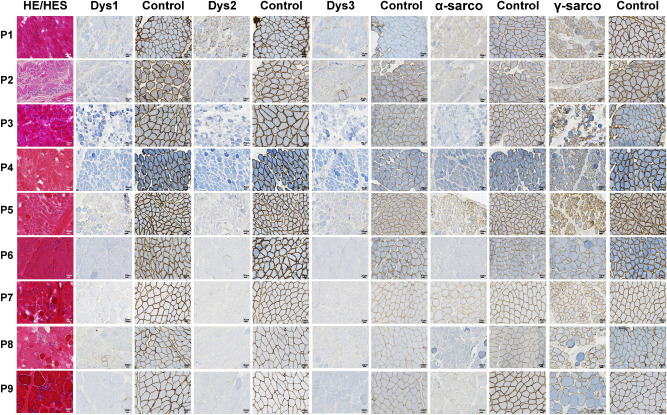
Morphological and immunohistochemical characteristics of DMD patients’ muscles. Left column: Haematoxylin–Eosin (HE) (P1, P3, P4, P5, P6, P7, P8, P9) or Hemalun-Eosin-Safran (HES) (P2) displaying endo-and perimysial fibrosis, necrotic and atrophic fibres along with inflammatory infiltrates suggestive of progressive musclar dystrophy. Second to seventh columns : dystrophin immunolabeling (dys 1, dys 2 and dys 3 labeling the core, NH2-terminal and COOH-terminal domains of the protein respectively) showing severely decreased or absent immunoreactivity in patients compared to control muscle apposed on the same slide. Eighth to eleventh columns : α- and γ-sarcoglycan immunolabelings showing either a decrease or normal immunoreactivity in patients compared to muscle control opposed on the same slide. Horizontal lines correspond to patients 1–9; scale bars: 20 µ.

Morphological lesions meeting DMD diagnostic criteria were observed in muscle biopsies including fiber necrosis with inflammatory response, diffuse variation in fiber size with rounded and hypercontracted fibers, basophilic regenerative fibers, and endomysial and perimysial fibrosis. Immunohistochemical studies revealed that six patients lacked DYS1, and three had severely decreased and irregular DYS1 immunoreactivity, all patients but one having no DYS2 and DYS3 (Fig. 1, Supplementary Tables 1 and 2). These expression patterns were confirmed by western blot analyses. Immunohistochemical or western blot techniques showed that absent or decreased DYS was associated with absent or decreased proportions of α-, β-, γ- and δ sarcoglycans (Fig. 1, Supplementary Tables 1 and 2). Molecular analyses allowed for identifying different variants consisting of large deletions in the DMD gene sequence in 8 patients and a small frameshift deletion in one patient (Supplementary Tables 1 and 2).

### Mass spectrometry-based metabolomics imaging

**Figure 2:**
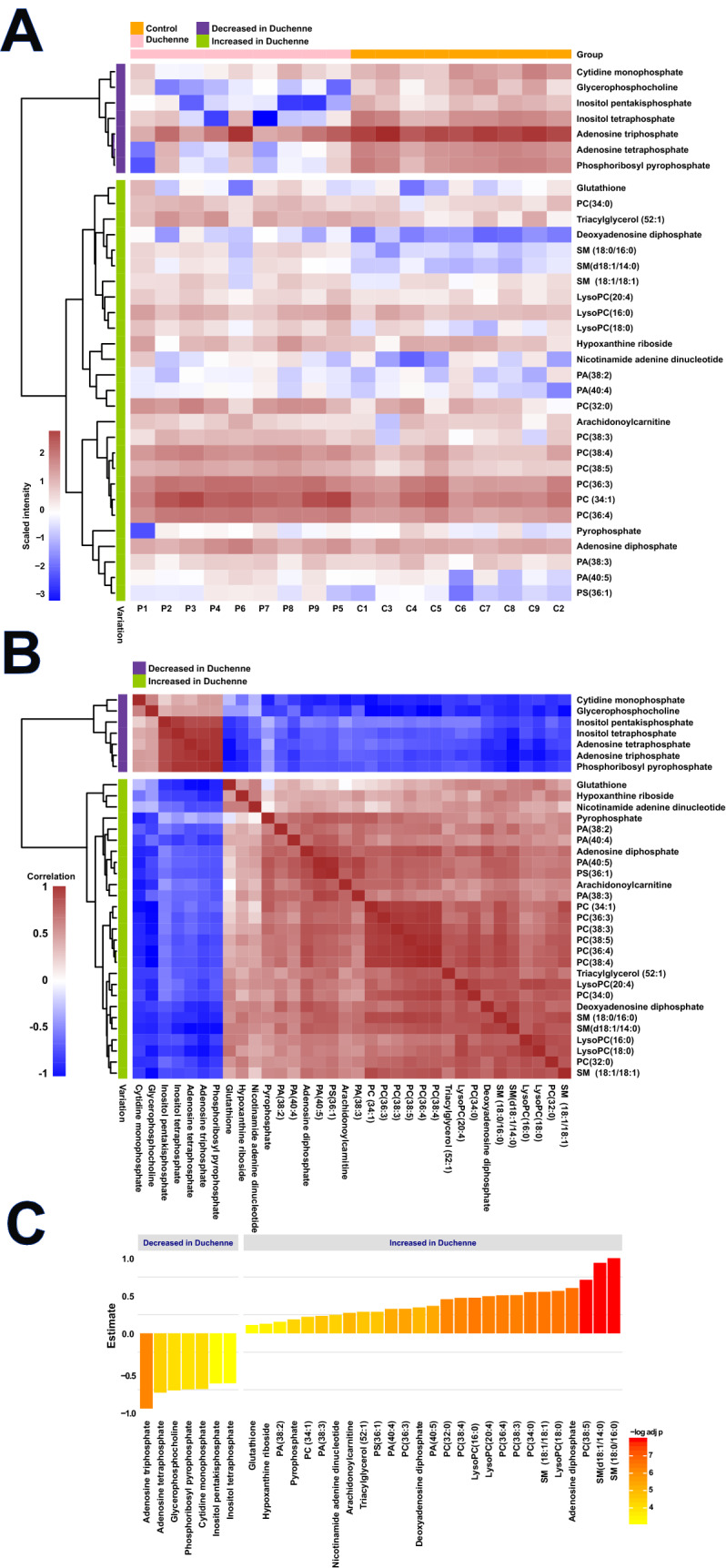
Metabolome variations between DMD and control tissues. (**A**) Unsupervised hierarchical clustering based on metabolic profiles (34 metabolites) detected in 9 DMD and 9 control tissues. C: Control, P: Patient; (**B**) Spearman correlation heatmap of the 34 differentially expressed metabolites between control and DMD tissues; (**C**) Barplots of the differentially expressed metabolites and their directional changes in the DMD tissues. Bar color is proportional to –log (adjusted p-value). Red denotes high significance.

**Figure 3:**
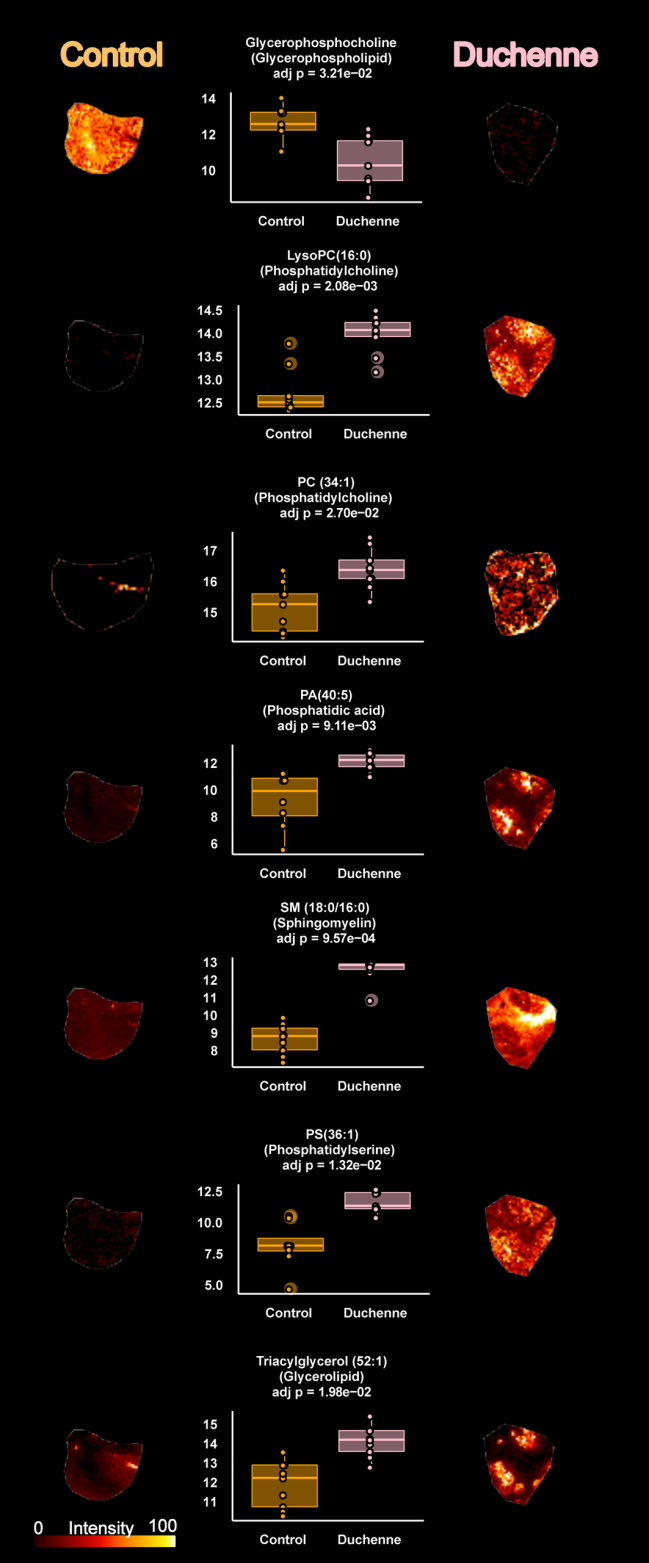
Tissue distribution of selected discriminant metabolites. Left) Tissue metabolite distribution in the control tissue. Right) Tissue metabolite distribution in the DMD tissue. Middle) Boxplot of the selected metabolites between DMD and control tissues with related adjusted p-values. The y-axis shows the log-scaled average intensity. Ion images were generated using SCiLs Lab software.

This work explored the differential metabolic patterns between Duchenne and control biopsies by analyzing the spectral fingerprints extracted in MSI experiments from the analyzed muscle biopsies. These fingerprints were formed from ions generated by the mass spectrometry analysis used for data analysis and metabolite identification. Average spectra are presented in Supplementary Fig. 1 The first statistical analysis yielded 52 discriminant ions with significant differences between the Duchenne and control biopsies. Further annotation steps allowed filtering and cleaning this list to include only unambiguously identified metabolites. In positive-ion mode, the combination of high mass accuracy and MS/MS experiments highlighted phosphatidylcholine (PC) and sphingomyelin (SM) lipids due to their fragmentation patterns. PC and SM lipids can be detected in positive-ion mode owing to their quaternary amine groups. The fragmentation ions observed upon collisional activation of both [M + Na]^+^ and [M + K]^+^ of PC and SM lipids included the loss of trimethylamine (59.073499 Da) and phosphocholine (183.066045 Da). Supplementary Fig. 2A shows the fragmentation pattern of C_28_H_50_NO_7_P at *m/z* 566.32166 [M + Na]^+^. Given the FTICR high mass accuracy, a loss of *m/z* 59.07351 and *m/z* 183.06608 corresponded to trimethylamine and phosphocholine with mass errors of 186 ppb and 191 ppb, respectively. Therefore, the ion at *m/z* 566.32166 with the molecular formula, C_28_H_50_NO_7_P, was attributed to lysoPC(20:4) [M + Na]^+^. Other PC and SM lipids were similarly unambiguously assigned. In negative-ion mode, other lipids included phosphatidic acid and phosphatidylserine. Adenosine triphosphate (ATP) at *m/z* 505.98833 was annotated using MS/MS analysis by the loss of an adenine group at *m/z* 272.95698 (C_5_H_8_O_9_P_2_, [M-H]^-^ with an error of 362 ppb; Supplementary Fig. 2B). Other metabolites and lipids were similarly assigned with the mass accuracy. The final list included 34 unambiguously identified features with 21 and 13 in positive- and negative-ionization modes, respectively. Supplementary Table 3 presents the data matrix; Supplementary Table 4 presents the related statistics of the identified metabolites along with their annotation metrics. Supplementary Fig. 3 presents the boxplots of the different metabolites. More mass spectra are presented in Supplementary Figs. 4–10. Seven metabolites were decreased in the Duchenne biopsies: adenosine tetraphosphate, adenosine triphosphate, cytidine monophosphate, glycerophosphocholine, inositol pentakisphosphate, inositol tetraphosphate, and phosphoribosyl pyrophosphate. The other 27 metabolites were increased in the Duchenne biopsies, including SM, phosphatidylcholines, phosphatidic acids and phosphatidylserines. To explore the distribution of this signature and its expression across samples, we performed a clustering analysis using Euclidean distance, a similarity metric, between samples. Figure 2A shows two distinct clusters between the DMD and control biopsies due to the differential expression of the above-mentioned metabolites across samples. We also explored the covariation of these metabolites using Spearman correlation analysis. Figure 2B shows two main co-expression clusters that include upregulated and downregulated metabolites. Intraclass subclusters are observed such as a module that includes phosphatidylcholines, phosphatidic acids and phosphatidylserines. Another module includes SMs and lysophosphatidylcholines. Figure 2C presents the directional changes of these metabolites in the DMD samples along with their statistical significance. This figure reports adjusted p-values and estimate which indicates metabolite change direction. Figure 3 presents boxplots of the glycerophosphocholine, lysophosphatidylcholine, phosphatidylcholine, phosphatidic acid, SM, phosphatidylserine, along with their tissue distributions in the DMD and control biopsies with their related adjusted p-values. Even though the figures don’t show a cellular, sub-cellular or fiber level resolution, they highlight the heterogenous distribution of intensities across the tissue section that mirrors the distribution of the related metabolites.

**Figure 4:**
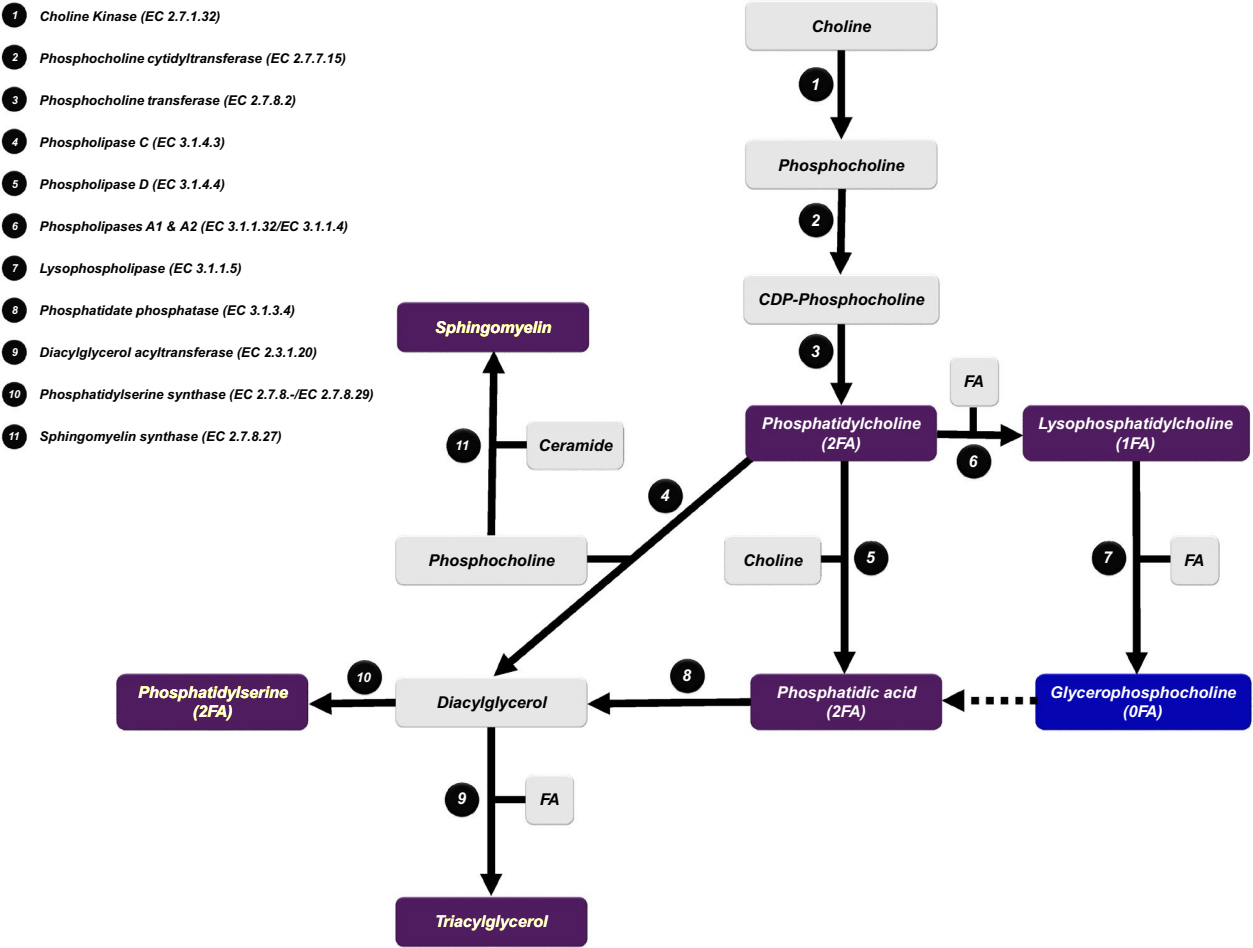
Phospholipid and triacylglycerol metabolism. Purple: increased metabolites in DMD. Blue: decreased metabolites in DMD. EC: Enzyme Commission number. FA: fatty acids.

## Discussion

Defective dystrophin is known to be the main alteration in the DMD etiology, however, metabolic impairment has been reported in several tissues such as the skeletal and cardiac muscles, liver and brain^27^. Here, we explored the differential metabolic patterns of muscle biopsies from DMD patients and control samples using a hypothesis-free strategy based on MSI. Our results showed profound metabolic pathway remodelling in the muscles of DMD patients compared with the controls grouped in two distinct clusters corresponding to the upregulated and downregulated metabolites. The most discriminative metabolites were primarily phospholipids and energy metabolites, suggesting that both these metabolic pathways are important players in muscle pathology and possibly by extension cognitive/behavioral disabilities.

Phospholipids (PLs) are major components of all cell membranes, and glycerophospholipids (GLs) are the most abundant membrane PLs. GLs are composed of a glycerol backbone linked to a phosphate group in position 3. Acyl groups may be attached at positions 1 and 2 of the glycerol. Different moieties, such as choline, inositol, ethanolamine and serine, are linked to the phosphate group, contributing to GL diversity (e.g., PC, phosphatidylinositol [PI], phosphatidylethanolamine (PE), PS, and phosphatidylglycerol). PCs are the most abundant GL in mammalian cells. Other lipid classes, such as cholesterol and glycosphingolipids, play structural roles in cell membranes^28,29^. Disruption of PL metabolism has been previously reported in DMD^10,11,13,20,21,23,30,31^. We showed that several compounds belonging to PC, lysophosphatidylcholine (LPC), phosphatidic acid (PA), PS and SM classes, as well as triacylglycerols, are increased, while glycerophosphocholine (GPC) is decreased in DMD muscles compared with control muscles (Fig. 3). This metabolic remodelling could be attributed to a putative decrease in lysophospholipase activity (Fig. 4). Lysophospholipase catalyzes lysophosphatidylcholine deacylation and mediates GPC production^32^. Sharma et al. considered DMD to be mainly a GPC deficiency^33^. Consequently, this impairment could lead to an accumulation in phosphatidylcholine, which is redirected toward other metabolic pathways with an increase in PA, SM, phosphatidylserine and triacylglycerol (Fig. 4). It has been shown that lysophospholipase activity in erythrocyte membranes of DMD patients was lower than that of healthy age-matched subjects^32^. Chalovich et al. compared the^31^P-NMR spectra of muscles from DMD patients, Werdnig-Hoffman (Spinal Muscular Atrophy Type 1) patients and controls. These authors observed that GPC levels were lower or near absent in DMD muscles compared with control muscles and dramatically increased (15-fold) in one Werdnig-Hoffman patient^30^. Increased triacylglycerol and SM in DMD mouse muscles have been interpreted as resulting from increased phospholipase C activity^13^. High phospholipid levels and high phospholipid-to-cholesterol ratios have been documented using^1^H-NMR spectroscopy, which helps distinguish DMD patients from healthy subjects^34^. Phospholipase D activity is reported to be increased in DMD muscles. Calcium homeostasis has been shown to be altered in DMD patients, with calcium concentration being increased in dystrophin-deficient muscle. Phospholipase D activity is enhanced under high concentrations of free calcium, resulting in increased PA levels^35,36^. In summary, profound phospholipid metabolism remodelling is associated with DYS deficiency; thus, DMD has been considered a glycerophosphocholine deficiency^33,37^. Morphological alterations of the mitochondria have been reported in DMD muscles with higher rates of swollen mitochondria compared with that of normal muscle^38^. PL alterations in DMD and a dysregulation of Ca^2+^ homeostasis may induce mitochondrial membrane fragility and associated morphological changes with subsequent energy and oxidative metabolism disruptions^39–42^.

Energy metabolism impairments in DMD are major contributors to DYS-deficient muscle degeneration^43^ as illustrated by mitochondrial function improvement upon the partial restoration of DYS expression^44^. Accordingly, we showed a significant increase in ADP and decreased ATP levels in DMD muscles compared with those of the control muscles (Supplementary Fig. 3). Several preclinical and clinical studies aimed at promoting energy metabolism are ongoing to treat DMD^45^. The need for studies with higher cellular or fiber level resolution would unveil deeper metabolic insights and further explore these results. Van Pelt et al. performed a multi-omics study in a Duchenne mouse model. They reported the impairment of glycolytic metabolism and phospholipids^46^. Two metabolites overlap with our results adenosine diphosphate and PC(36:3) (Supplementary Table 5). Exploring the potential release of the metabolites in less invasive biological fluids is relevant. Spitali et al. reported serum-based metabolomics results in patients affected by several multiple forms of muscular dystrophy. Fifteen metabolites have been reported belonging to energy, amino acid and testosterone metabolisms^47^. Lindsay et al. performed a urine-based metabolomics study in a Duchenne mouse model. They reported that five of seven detected Krebs cycle metabolites were depleted in these mice consistent with an impaired energy metabolism^22^. No overlap has been observed between our results and those reported in these studies (Supplementary Table 5).

In conclusion, although metabolic impairments have been reported in DMD, this work describes for the first time the use of ultra-high-resolution MSI in DMD, which enabled more systematically and integratively exploring on-tissue metabolic disturbances. This powerful technique allowed investigating the different metabolic components in a single experiment. The present results highlight the potential use of MSI technology coupled with systems biology approaches to holistically explore metabolic impairments in DMD. This work lays the foundation for more mechanistic investigations of DMD and other metabolic diseases.

## Methods

The overall workflow is presented in Supplementary Fig. 11.

### Patients

Nine patients with molecularly proven DMD over the last 10 years were selected for the study. All patients were referred to our neurology department, and biopsies were performed as a part of the diagnostic workup when the disease was suspected. Samples were collected before initiating treatment. Medical charts were reviewed for age at disease onset, age at diagnosis, age at walking unaided, age at walking loss, and age at last visit. Symptoms at disease onset were recorded as calf hypertrophy, muscle weakness, contractures, behavioral problems, speech delay, respiratory distress and cardiac arrhythmia. Disease activity parameters consisted of maximal motor function, maximal CPK, age of initial joint contractures and current joint contractures, surgical therapy and long-term physical therapy. Other parameters consisted of scoliosis occurrence or any other spinal deformities, along with their surgical and nonsurgical therapies, occurrence of bone fractures and other orthopedic surgeries, and mobility at last visit. Evaluated complications were respiratory insufficiency (e.g., IPPB, ventilation, tracheotomy), existing cardiac complications (e.g., rhythm abnormalities or heart abnormalities on ultrasound), digestive complications (e.g., nutritional problems, gastrostomy) and iatrogenic complications. Inclusion in therapeutic protocols, hospitalization frequency, cognitive delays and behavioral problems, including autism and school attendance, were also recorded.

**Figure 5:**
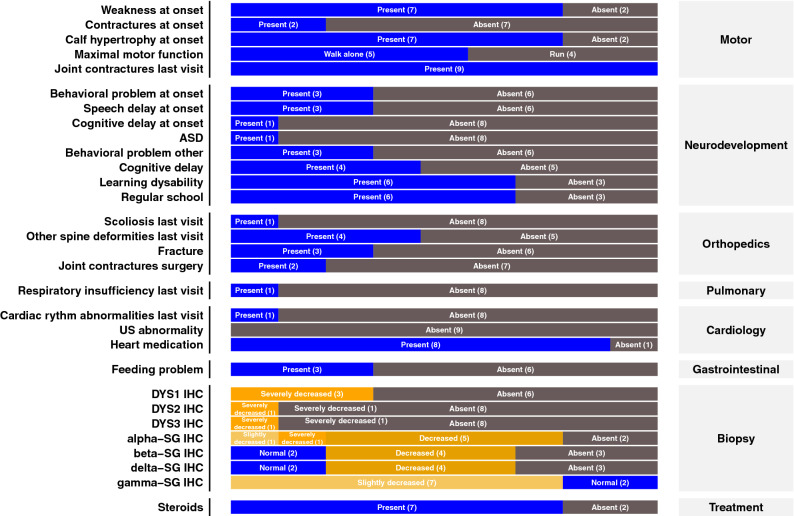
Clinical characteristics of DMD patients. ASD: autistic spectrum disorders, DYS: dystrophin, IHC: immunohistochemistry, US: ultrasound, SG: sarcoglycan.

The mean patient age at disease onset was 4 ± 1.5 years, at last visit was 10.5 ± 2.2 years, and at time of biopsy was 5.5 ± 1.9 years. All patients are presently alive, and six (P1, P5, P6, P7, P8, and P9) were ambulant at the last visit. One non-ambulant patient (P2) lost the ability to walk at age 7; he was not given steroids, nor included in any therapeutic protocol. The other two patients (P3, P4) lost the ability to walk at 11 and 13 years of age. CK levels ranged from 5418–52,000. Symptoms at disease onset were muscle weakness with difficulty walking and climbing stairs and frequent falls. Eight patients had calf hypertrophy. Six patients presented no motor features at onset but had speech delays (P2, P5, P8) and/or behavioral/autistic disturbances (P5, P8, P9) that had likely led to delayed diagnosis. Four patients had cognitive delays (P2, P5, P8, P9). Two patients (P2, P5) developed behavioral abnormalities. Six patients had learning difficulties affecting speech, writing, attention and memory (P1, P2, P5, P7, P8, P9). Three patients (P4, P5, P9) had nutritional and feeding problems, with anorexia in two and obesity in one (P9). All but two patients were given steroids (P1, P2); one of these two patients were lost to follow up one year after diagnosis, and the other received no treatment owing to poor family compliance. Four patients (P1, P5, P7, P9) could run, the others could walk without help. Two patients had mild respiratory insufficiency that did not require ventilation. Eight patients had no significant cardiac arrhythmia or functional abnormalities. Patient P4 had rhythm abnormalities consisting of supraventricular extrasystoles 3.9% of the time, which did not require specific treatment. All but one patient received a prophylactic antiarrhythmic drug (perindopril), and one (P1) was included in a perindopril protocol (2-year double-blind treatment protocol). Three patients (P4, P5, P7) were included in exon-skipping protocols, and one (P6) was included in a givinostat (histone deacetylase inhibitor) protocol. Clinical data overview is presented in Fig. 5 and detailed clinical data are listed in Supplementary Tables 1 and 2.

### Muscle biopsies

Each patient underwent a muscle biopsy at a mean age of 5.5 ± 1.9 years, which was assessed according to standardized histochemical and histoenzymological methods^48^. For immunohistochemical studies, 6-μm frozen sections were immunolabeled with antibodies against β-spectrin (diluted 1:50; Novocastra Leica Biosystems, Nanterre, France), dystrophin (DYS1, DYS2 and DYS3 correspond to amino acids 1181–1388, 3669–3685 and 321–494, of the dystrophin molecule and are diluted respectively at 1:10, 1:8 and 1:6—Novocastra Leica Biosystems), and α-, β-, γ- and δ sarcoglycans (diluted 1:100, 1:100, 1:100 and 1:50, respectively; Novocastra Leica Biosystems). Dystrophinopathy was morphologically diagnosed per the criteria of Dubowitz et al.^48^.

As age-matched control muscles were not available, nine male adults aged 28–58 years were selected as controls for comparative analyses. These patients presented myalgias, muscle weakness, and/or muscle fatigability. In one case, a polymyositis was suspected; Buschke scleroderma was suspected in another. In all control patients, routine biological tests, CK levels, EMGs, cardiac ultrasonography, pulmonary testing and muscle MRI were considered normal. Their muscle biopsies were also normal, and they were concluded to have probable fibromyalgia. One obvious potential limit of this work that needs to be highlighted consists in absence of age-matched control muscles could hamper interpretation of the results, as biochemical properties of muscles change over the lifetime. Even though paediatric muscle samples could be obtained during orthopaedic surgery, the location of the biopsy may change and may not entirely match with that of Duchenne patients.

### Reagents and chemicals

Methanol, acetonitrile, ethanol and water of LC–MS grade were purchased from Fisher Scientific (Loughborough, UK); 2.5-dihydroxybenzoic acid (DHB) and 9-aminoacridine hydrochloride monohydrate (9-AA) MALDI matrices were purchased from Sigma-Aldrich (St. Louis, MO, USA). Sodium trifluoroacetate (NaTFA, Sigma-Aldrich) at 0.1 mg mL^-1^ (ACN/H_2_O 50:50 [v/v]) was used as a calibrant before each analysis.

### Sample preparation

Fresh frozen 10-µm sections of human muscle were mounted on conductive ITO-coated glass slides 75 × 25 mm (Bruker, Bremen, Germany) and stored at -80 °C until analysis. Matrix solutions were applied with an automatic microsprayer HTX TM-Sprayer (HTX Imaging, Chapel Hill, NC, USA) as previously described^49^. In the positive-ion mode, DHB matrix (30 mg mL^-1^ in MeOH/H_2_O 50:50 [v/v]) was deposited with the following parameters: nozzle temperature 80 °C, nozzle velocity 1200 mm min^-1^, N_2_ pressure 10 psi, N_2_ flow rate 2 L min^-1^, number of passes 12, flow rate 100 µL min^-1^ and track spacing 3 mm. In negative-ion mode, 9-AA matrix (10 mg mL^-1^, EtOH/H_2_O 70:30 [v/v]) was sprayed using the following parameters: nozzle temperature 90 °C, nozzle velocity 1200 mm min^-1^, N_2_ pressure 10 psi, N_2_ flow rate 3 L min^-1^, number of passes 2, flow rate 120 µl min^-1^, track spacing 3 mm and drying time 30 s between passes. Each slide was vacuum-dried before analysis.

### Mass spectrometry instrumentation and data processing

Data were acquired on a FTICR instrument (SolariX XR, Bruker, Bremen, Germany) equipped with a 12-T superconducting magnet and a dynamically harmonized ICR cell. This instrument is also equipped with both a laser desorption ionization source (Smartbeam II, Nd:YAG × 3 laser at 355 nm, Bruker, Bremen, Germany) and an electrospray (ESI) source. Each MALDI spectrum for each position is the result of 1 scan and 500 consecutive laser shots. Spectra were acquired over an 80 µm raster. Before imaging analyses, the instrument was externally calibrated in the required mode by NaTFA infusion via ESI source, then internally calibrated by assigning known metabolites from m/z 150–1000 via MALDI source. The instrument was autocalibrated during image acquisition. In positive-ionization mode, calibration was performed by assigning C_7_H_6_O_4_ (*m/z* 155.033885 [M + H]^+^, matrix peak), C_7_H_15_NO_3_ (*m/z* 162.112470 [M + H]^+^, carnitine), C_7_H_6_O_4_ (*m/z* 177.015829, [M + Na]^+^, matrix peak), C_5_H_14_NO_4_P (*m/z* 184.073321, [M + H]^+^, phosphocholine), C_9_H_17_NO_4_ (*m/z* 204.123034, [M + H]^+^, acetylcarnitine), C_14_H_8_O_6_ (*m/z* 273.039364, [M + H]^+^, matrix peak), C_21_H_12_O_9_ (*m/z* 409.055408, [M + H]^+^, matrix peak), C_40_H_80_NO_8_P (*m/z* 734.569432, [M + H]^+^, lipid), and C_42_H_82_NO_8_P (*m/z* 798.54096, [M + K]^+^, lipid). In negative-ion mode the assigned peaks were C_13_H_10_N_2_ (*m/z* 193.77122, [M-H]^-^, matrix peak), C_17_H_26_N_6_O_6_ (*m/z* 409.184106, [M-H]^-^, amino acids), C_10_H_15_N_5_O_10_P_2_ (*m/z* 426.022139, [M-H]^-^, adenosine diphosphate), C_10_H_16_N_5_O_13_P_3_ (*m/z* 505.988470, [M-H]^-^, adenosine triphosphate), C_23_H_39_N_7_O_7_ (*m/z* 524.283820, [M-H]^-^, amino acids), C_30_H_46_N_6_O_8_ (*m/z* 599.319871, [M-H]^-^, lipid) and C_39_H_77_O_9_P (*m/z* 701.512680, [M-H]^-^, lipid). Data size was set at 2 million points for a transient length of 0.87 s, and spectra were acquired with a 97% data file reduction. A single MSI measurement has been performed by specimen. Images were captured using FTMS control and FlexImaging (v 5.0, Bruker) software. Images were processed with SCiLS Lab Pro software (Bruker Daltonics, Bremen, Germany). The total ion current method was used for normalization, and m/z intervals were automatically set at ± 1 ppm. Images were viewed using both FlexImaging and SCiLS Lab software (Bruker Daltonics, Bremen, Germany).

### Data analyses

Ion intensities have been log-transformed. Univariate analyses were performed using t-tests to identify discriminatory ionic species between the assessed groups. Age has been taken into account by adding it as covariate. Spearman correlation analysis was performed using R software. Euclidean distance was used as a similarity measure in the clustering analysis. False discovery rates were corrected using the Benjamini–Hochberg–Yekutieli method, and p < 0.05 was considered statistically significant.

### Metabolite annotation and identification

Preliminary assignments based on accurate mass measurements were performed using the mass spectrometry databases, METLIN ^50^ and HMDB ^51^, using a threshold of ± 2 ppm. For some metabolites, the precise raw formula led to one hit. Others were identified via “on-tissue” tandem mass spectrometry experiments using MALDI tandem MS/MS. Ions of interest were first isolated using a window of ± 1 Da, then fragmented by collision-induced dissociation with energy levels between 10 and 40 eV. For each MS/MS analysis, 50 scans were accumulated for better sensitivity. Spectra were reprocessed using Data Analysis 4.4 software (Bruker Daltonics, Bremen, Germany) and recalibrated with the single-point calibration option.

### Ethical statement

All muscle samples used in this study belong to a collection declared to the French Ministry of Health (collection number DC-2015–2468, accession number AC-2015–2467) located in the Pathology Department (Prof. Annie Laquerrière) of Rouen University Hospital in accordance with the relevant guidelines and regulations and with permission of the local authorities. Written informed consents were obtained from the parents when the patient is under 18 or from the adult patient in order to perform any investigation related to their pathology. The study protocols have been approved by ethics committee of NORD WEST1 – Rouen University Hospital (collection number DC-2015–2468, accession number AC-2015–2467).

## Supplementary information

Supplementary Tables.

Supplementary Figures.

## Data Availability

All the data that support the findings are presented in the manuscript and the supplementary material.

## References

[1] Al-Khalili Szigyarto, C., & Spitali, P. Biomarkers of Duchenne muscular dystrophy: current findings. *Degenerative Neurological and Neuromuscular Disease* **8**, 1–13, 10.2147/dnnd.s121099 (2018). 10.2147/DNND.S121099 PMC6053903 30050384

[2] Birnkrant DJ (2018). Diagnosis and management of Duchenne muscular dystrophy, part 1: diagnosis, and neuromuscular, rehabilitation, endocrine, and gastrointestinal and nutritional management. The Lancet. Neurol..

[3] Mercuri E, Bonnemann CG, Muntoni F (2019). Muscular dystrophies. Lancet.

[4] Guiraud S (2015). The pathogenesis and therapy of muscular dystrophies. Annu. Rev. Genomics Hum. Genet..

[5] Guiraud S, Davies KE (2019). Regenerative biomarkers for Duchenne muscular dystrophy. Neural Regen. Res..

[6] Doorenweerd N (2017). Timing and localization of human dystrophin isoform expression provide insights into the cognitive phenotype of Duchenne muscular dystrophy. Sci. Rep..

[7] Signorelli, M. *et al.* Longitudinal serum biomarker screening identifies malate dehydrogenase 2 as candidate prognostic biomarker for Duchenne muscular dystrophy. *J. Cachexia, Sarcopenia Muscle*, 10.1002/jcsm.12517 (2019). 10.1002/jcsm.12517 PMC7113516 31881125

[8] Hoxha, M. Duchenne muscular dystrophy: Focus on arachidonic acid metabolites. *Biomed. Pharmacother. 10*, 796–802, 10.1016/j.biopha.2018.12.034 (2019). 10.1016/j.biopha.2018.12.034 30554118

[9] Muntoni F (2019). Categorising trajectories and individual item changes of the north star ambulatory assessment in patients with Duchenne muscular dystrophy. PLoS ONE.

[10] Chio C, Peterson D, Kratzer F (1972). Lipid composition and synthesis in the muscles of normal and dytrophic chickens. Can. J. Biochem..

[11] Hughes BP (1972). Lipid changes in Duchenne muscular dystrophy. J. Neurol. Neurosurg. Psychiatry.

[12] Kwok CT, Kuffer AD, Tang BY, Austin L (1976). Phospholipid metabolism in murine muscular dystrophy. Exp. Neurol..

[13] Kwok CT, Austin L (1978). Phospholipid composition and metabolism in mouse muscular dystrophy. Biochem. J..

[14] Tebani, A., Afonso, C., Marret, S. & Bekri, S. Omics-Based Strategies in Precision Medicine: Toward a Paradigm Shift in Inborn Errors of Metabolism Investigations. *International journal of molecular sciences* **17**, 10.3390/ijms17091555 (2016). 10.3390/ijms17091555 PMC5037827 27649151

[15] Nicholson JK, Lindon JC, Holmes E (2008). 'Metabonomics': understanding the metabolic responses of living systems to pathophysiological stimuli via multivariate statistical analysis of biological NMR spectroscopic data. Xenobiotica For. Compound. Biol. Syst..

[16] Tebani, A., Afonso, C. & Bekri, S. Advances in metabolome information retrieval: turning chemistry into biology. Part I: analytical chemistry of the metabolome. *J Inherit Metab Dis*, 10.1007/s10545-017-0074-y (2017). 10.1007/s10545-017-0074-y PMC5959978 28840392

[17] Spengler B (2015). Mass spectrometry imaging of biomolecular information. Anal. Chem..

[18] Zhang, J. *et al.* Nondestructive tissue analysis for ex vivo and in vivo cancer diagnosis using a handheld mass spectrometry system. *Sci Transl Med* **9**, 10.1126/scitranslmed.aan3968 (2017). 10.1126/scitranslmed.aan3968 PMC5830136 28878011

[19] Xiao, Y. *et al.* Recent advances of ambient mass spectrometry imaging for biological tissues: A review. *Analytica Chimica Acta* (2020). 10.1016/j.aca.2020.01.052 32408956

[20] Benabdellah F, Yu H, Brunelle A, Laprevote O, De La Porte S (2009). MALDI reveals membrane lipid profile reversion in MDX mice. Neurobiol Dis.

[21] Touboul D (2004). Changes of phospholipid composition within the dystrophic muscle by matrix-assisted laser desorption/ionization mass spectrometry and mass spectrometry imaging. Eur. J. Mass Spectrom. (Chichester, England).

[22] Lindsay A, Chamberlain CM, Witthuhn BA, Lowe DA, Ervasti JM (2019). Dystrophinopathy-associated dysfunction of Krebs cycle metabolism. Hum. Mol. Genet..

[23] Tahallah N, Brunelle A, De La Porte S, Laprevote O (2008). Lipid mapping in human dystrophic muscle by cluster-time-of-flight secondary ion mass spectrometry imaging. J. Lipid Res..

[24] Shaw JB (2016). 21 Tesla Fourier transform ion cyclotron resonance mass spectrometer greatly expands mass spectrometry toolbox. J. Am. Soc. Mass Spectrom..

[25] Kooijman PC (2019). Increased throughput and ultra-high mass resolution in DESI FT-ICR MS imaging through new-generation external data acquisition system and advanced data processing approaches. Sci. Rep..

[26] Ferey J (2019). A new optimization strategy for MALDI FTICR MS tissue analysis for untargeted metabolomics using experimental design and data modeling. Anal. Bioanal. Chem..

[27] Esposito G, Carsana A (2019). Metabolic alterations in cardiomyocytes of patients with duchenne and becker muscular dystrophies. Journal of Clinical Medicine.

[28] Hishikawa D, Hashidate T, Shimizu T, Shindou H (2014). Diversity and function of membrane glycerophospholipids generated by the remodeling pathway in mammalian cells. J. Lipid Res..

[29] Vance JE (2015). Phospholipid synthesis and transport in mammalian cells. Traffic (Copenhagen, Denmark).

[30] Chalovich JM, Burt CT, Danon MJ, Glonek T, Barany M (1979). Phosphodiesters in muscular dystrophies. Ann N Y Acad Sci.

[31] Pearce P, Johnsen R, Wysocki S, Kakulas B (1981). Muscle lipids in Duchenne muscular dystrophy. Aust. J. Exp. Biol. Med. Sci..

[32] Podolski JL (1983). Erythrocyte membrane lysophospholipase activity in muscular dystrophy. J. Neurol. Sci..

[33] Sharma U, Atri S, Sharma MC, Sarkar C, Jagannathan NR (2003). Skeletal muscle metabolism in Duchenne muscular dystrophy (DMD): an in-vitro proton NMR spectroscopy study. Magn. Reson. Imaging.

[34] Srivastava NK, Pradhan S, Mittal B, Gowda GA (2010). High resolution NMR based analysis of serum lipids in Duchenne muscular dystrophy patients and its possible diagnostic significance. NMR Biomed..

[35] McDermott, M. I., Wang, Y., Wakelam, M. J. O. & Bankaitis, V. A. Mammalian phospholipase D: Function, and therapeutics. *Progress in Lipid Res.*, 101018, 10.1016/j.plipres.2019.101018 (2019). 10.1016/j.plipres.2019.101018 PMC7233427 31830503

[36] Walter M (2000). Involvement of phospholipase D in store-operated calcium influx in vascular smooth muscle cells. FEBS Lett..

[37] Infante JP, Huszagh VA (1999). Mechanisms of resistance to pathogenesis in muscular dystrophies. Mol. Cell. Biochem..

[38] Pellegrini C (2013). Melanocytes–a novel tool to study mitochondrial dysfunction in Duchenne muscular dystrophy. J. Cell. Physiol..

[39] Dubinin, M. V. *et al.* Duchenne muscular dystrophy is associated with the inhibition of calcium uniport in mitochondria and an increased sensitivity of the organelles to the calcium-induced permeability transition. *Biochimica et biophysica acta. Molecular basis of disease* **1866**, 165674, 10.1016/j.bbadis.2020.165674 (2020). 10.1016/j.bbadis.2020.165674 31926263

[40] Hughes MC (2019). Early myopathy in Duchenne muscular dystrophy is associated with elevated mitochondrial H2 O2 emission during impaired oxidative phosphorylation. J. Cachexia, Sarcopenia Muscle.

[41] Kuno A (2018). Resveratrol ameliorates mitophagy disturbance and improves cardiac pathophysiology of dystrophin-deficient mdx mice. Sci. Rep..

[42] Lin YF (2019). A novel mitochondrial micropeptide MPM enhances mitochondrial respiratory activity and promotes myogenic differentiation. Cell Death Disease.

[43] Timpani CA, Hayes A, Rybalka E (2015). Revisiting the dystrophin-ATP connection: How half a century of research still implicates mitochondrial dysfunction in Duchenne Muscular Dystrophy aetiology. Med. Hypotheses.

[44] Vila MC (2017). Mitochondria mediate cell membrane repair and contribute to Duchenne muscular dystrophy. Cell Death Differ..

[45] Rybalka E, Timpani CA, Stathis CG, Hayes A, Cooke MB (2015). Metabogenic and nutriceutical approaches to address energy dysregulation and skeletal muscle wasting in duchenne muscular dystrophy. Nutrients.

[46] Van Pelt, D. W. *et al.* Multiomics analysis of the mdx/mTR mouse model of Duchenne muscular dystrophy. *Connective Tissue Research*, 1–16, 10.1080/03008207.2020.1791103 (2020).

[47] Spitali P (2018). Cross-sectional serum metabolomic study of multiple forms of muscular dystrophy. J. Cell Mol. Med..

[48] Dubowitz V, Sewry C, Oldfors A (2013). Muscle Biopsy: A Practical Approach.

[49] Ferey J (2019). A new optimization strategy for MALDI FTICR MS tissue analysisfor untargeted metabolomics using experimental design and datamodeling. ABC.

[50] Smith CA (2005). METLIN: a metabolite mass spectral database. Ther. Drug Monit..

[51] Wishart, D. S. *et al.* HMDB 3.0--The Human Metabolome Database in 2013. *Nucleic Acids Res.* **41**, 801 (2013). 10.1093/nar/gks1065 PMC3531200 23161693

