# Supplementary material for: Muscle metabolic remodelling patterns in Duchenne muscular dystrophy revealed by ultra-high-resolution mass spectrometry imaging
Source: Sci Rep. 2021 Jan 21;11:1906. doi: 10.1038/s41598-021-81090-1 (PMC7819988; doi:10.1038/s41598-021-81090-1)
Supplement: Supplementary file 2 — Supplementary Figures. [file 41598_2021_81090_MOESM2_ESM.docx]

Supplementary Materials for

**Muscle metabolic remodelling patterns in Duchenne muscular dystrophy revealed by ultra-high resolution mass spectrometry imaging**

Ivana Dabaj^1,2#^, Justine Ferey^3#^, Florent Marguet^2,4^, Vianney Gilard^3,5^, Carole Basset^4^, Youssef Bahri^6^, Anne-Claire Brehin^7^, Catherine Vanhulle^1^, France Leturcq^8^, Stéphane Marret^1,2^, Annie Laquerrière^2,4^, Isabelle Schmitz-Afonso^6^, Carlos Afonso^6^, Soumeya Bekri^2,3*^, Abdellah Tebani^3^

^1^ Rouen University Hospital, Department of Neonatal Pediatrics, Intensive Care and Neuropediatrics, Rouen, 76031, France

^2^ Normandie Univ, UNIROUEN, CHU Rouen, INSERM U1245, 76000 Rouen, France

^3^ Rouen University Hospital, Metabolic Biochemistry Department, Rouen, 76031, France.

^4^ Rouen University Hospital, Department of Pathology, Rouen, France.

^5^ Rouen University Hospital, Department of Neurosurgery, Rouen, France.

^6^ Normandie Univ, COBRA UMR 6014 et FR 3038 Univ Rouen; INSA Rouen; CNRS IRCOF, 1 Rue TesnieÌre, 76821 Mont-Saint-Aignan Cedex France

^7^ Normandie Univ, UNIROUEN, Inserm U1245 and Rouen University Hospital, Department of Genetics and Reference Center for Developmental Disorders, F 76000, Normandy Center for Genomic and Personalized Medicine, Rouen, France

^8^ APHP, Laboratoire de Génétique et Biologie moléculaire, HUPC Cochin, Paris, France.

#these authors contributed equally to this work

***Corresponding author:**

Prof. Soumeya BEKRI

Department of Metabolic Biochemistry,

Rouen University Hospital 76031 ROUEN Cedex France

[soumeya.bekri@chu-rouen.fr](mailto:soumeya.bekri@chu-rouen.fr)

Tel 00 33 2 32 88 81 24

Fax 00 33 2 32 88 83 41

Other Supplementary Material provided as an Excel file for this manuscript includes the following:

**Supplementary Table 1.** Clinical data for 9 Duchenne patients included in this study.

**Supplementary Table 2.** Descriptive statistics of the clinical data.

**Supplementary Table 3.** Complete intensity table of the annotated metabolites in the analysis’s biopsies.

**Supplementary Table 4.** Complete statistical and annotation metrics of the included metabolites.

**Supplementary Table 5.** Metabolite comparison with reported literature.

**Supplementary Figure 1**. Average spectra from Duchenne and Control samples in both Positive and Negative ionization mode.


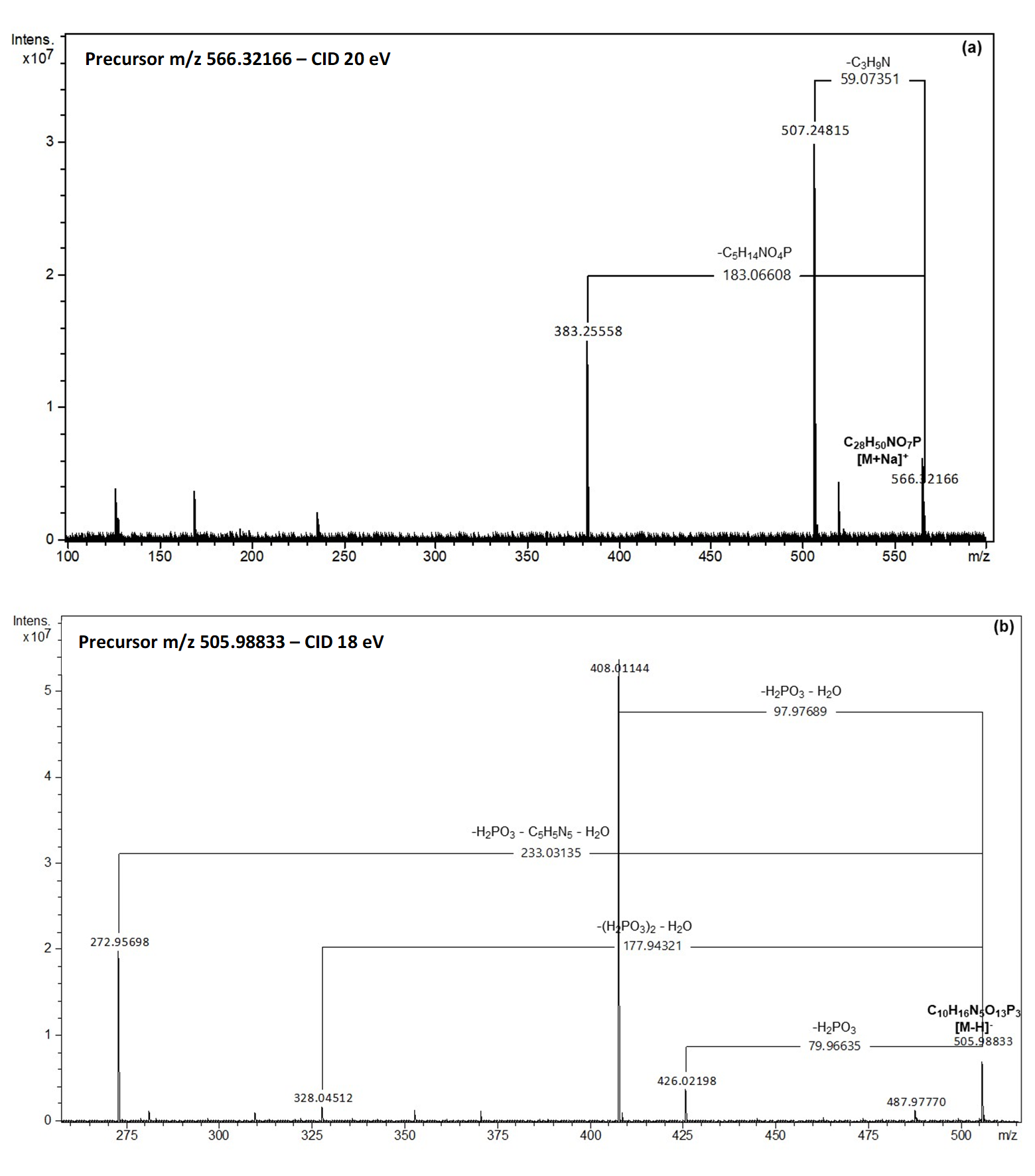


**Supplementary Figure 2**. MALDI-MS/MS spectra obtained from a) lysoPC(20:4) analyzed in positive ion mode by using DHB as a matrix and (b) Adenosine triphosphate (ATP) analyzed in negative ion mode by using 9-AA as a matrix. Spectra obtained using a Bruker SolariX MALDI-FTICR MS 12 Tesla. Collision induced dissociation (CID) was used for acquiring the MS/MS spectra.

**Supplementary Figure 3**. Boxplots of the differentially expressed metabolites between Duchenne and control biopsies**.**

**
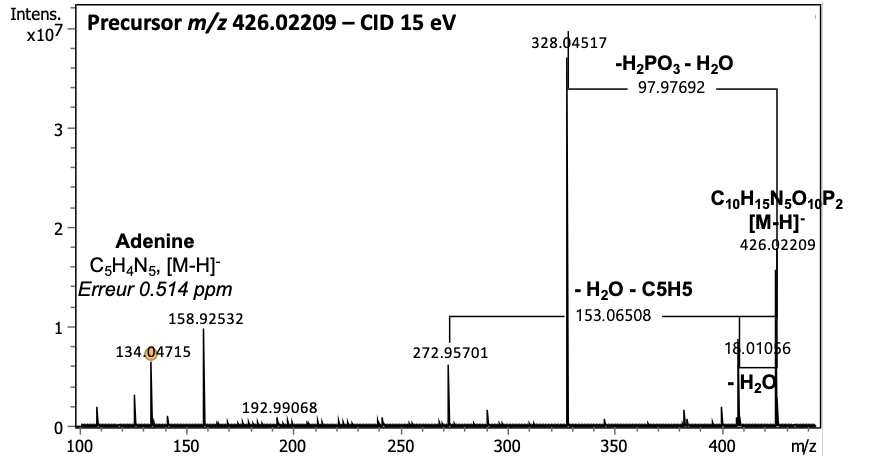
**

**Supplementary Figure 4**. MALDI-MS/MS spectra obtained from adenosine diphosphate (ADP, precursor *m/z* 426.02209, [M-H]^-^) analyzed in negative ion mode by using 9-AA as a matrix Spectra obtained using a Bruker solariX MALDI-FTICR MS 12 Tesla. Collision induced dissociation (CID) at 15 eV was used for acquiring the MS/MS spectra.

**
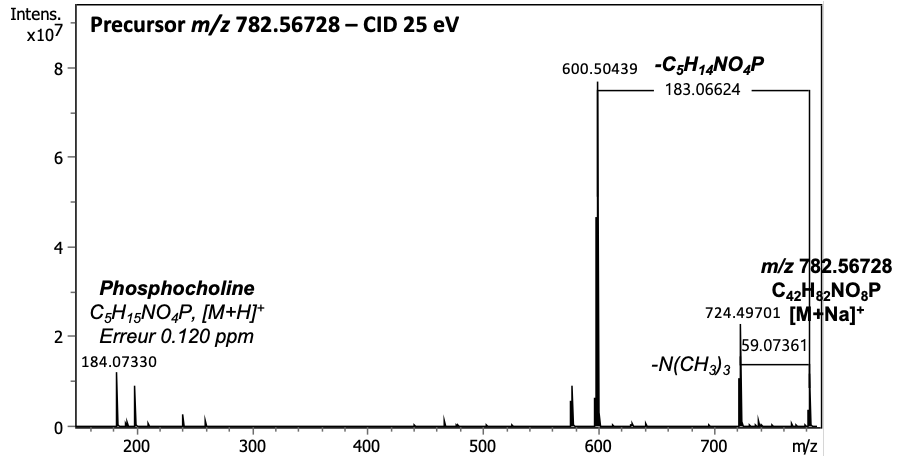
**

**Supplementary Figure 5**. MALDI-MS/MS spectra obtained from phosphatidylcholine 34:1 (PC 34:1, precursor *m/z* 782.56728, [M+Na]^+^) analyzed in positive ion mode by using DHB as a matrix Spectra obtained using a Bruker solariX MALDI-FTICR MS 12 Tesla. Collision induced dissociation (CID) at 25 eV was used for acquiring the MS/MS spectra.

**
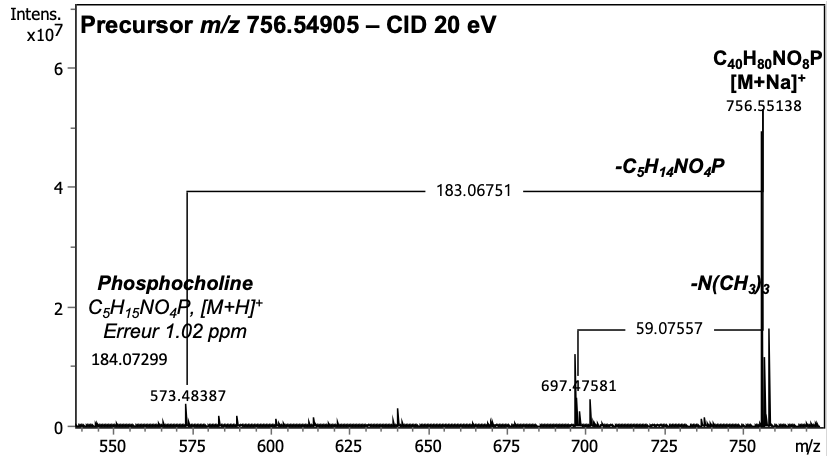
**

**Supplementary Figure 6**. MALDI-MS/MS spectra obtained from phosphatidylcholine 32:0 (PC 32:0, precursor *m/z* 756.55138, [M+Na]^+^) analyzed in positive ion mode by using DHB as a matrix Spectra obtained using a Bruker solariX MALDI-FTICR MS 12 Tesla. Collision induced dissociation (CID) at 20 eV was used for acquiring the MS/MS spectra.

**
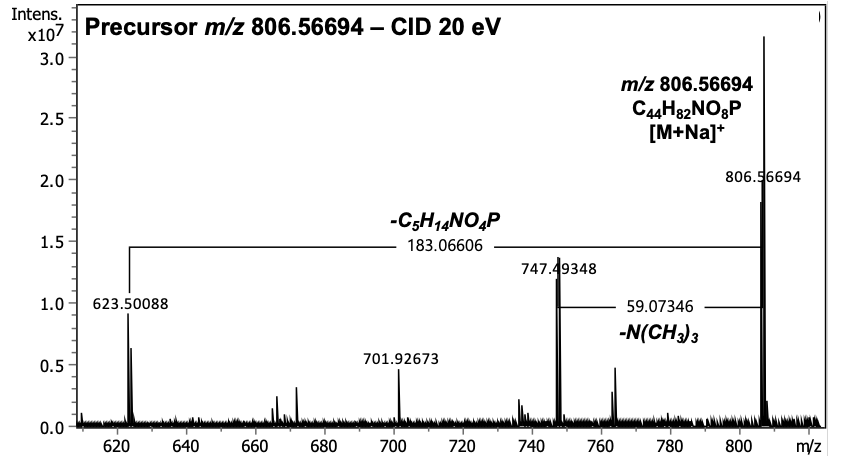
**

**Supplementary Figure 7**. MALDI-MS/MS spectra obtained from phosphatidylcholine 32:0 (PC 32:0, precursor *m/z* 756.55138, [M+Na]^+^) analyzed in positive ion mode by using DHB as a matrix Spectra obtained using a Bruker solariX MALDI-FTICR MS 12 Tesla. Collision induced dissociation (CID) at 20 eV was used for acquiring the MS/MS spectra.


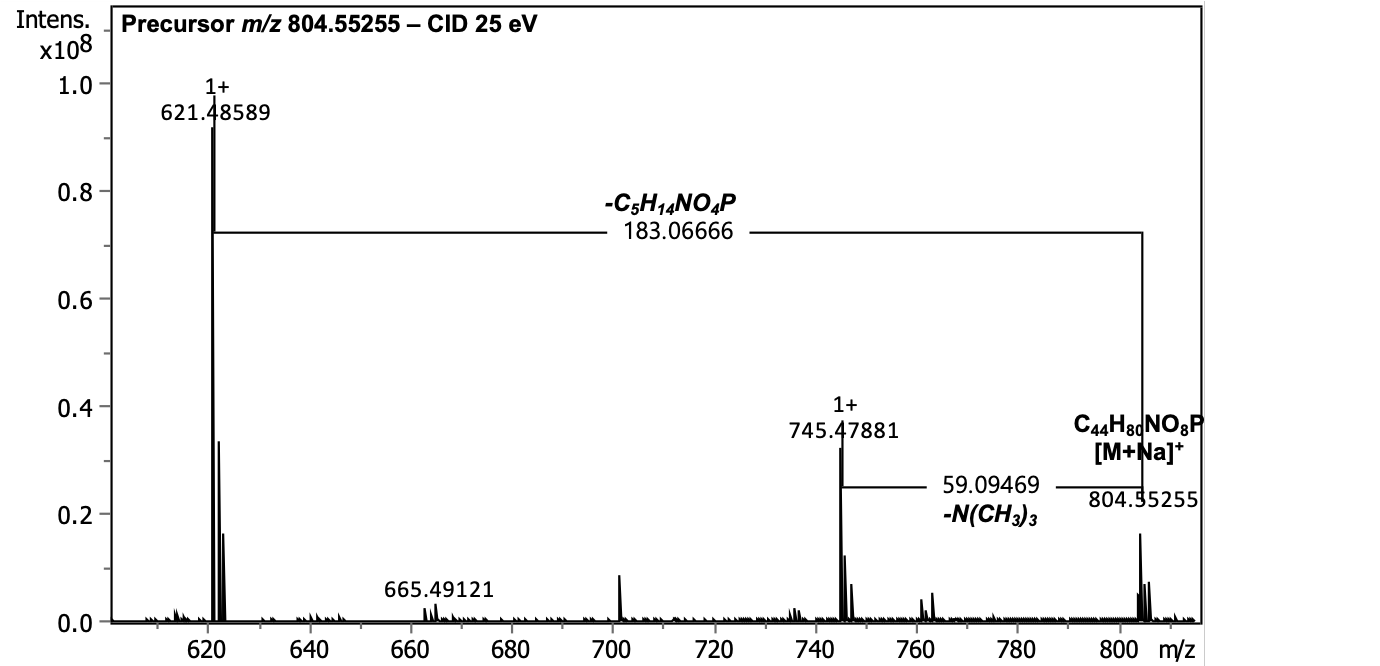


**Supplementary Figure 8**. MALDI-MS/MS spectra obtained from phosphatidylcholine 36:4 (PC 36:4, precursor *m/z* 804.55255, [M+Na]^+^) analyzed in positive ion mode by using DHB as a matrix Spectra obtained using a Bruker solariX MALDI-FTICR MS 12 Tesla. Collision induced dissociation (CID) at 25 eV was used for acquiring the MS/MS spectra.

**
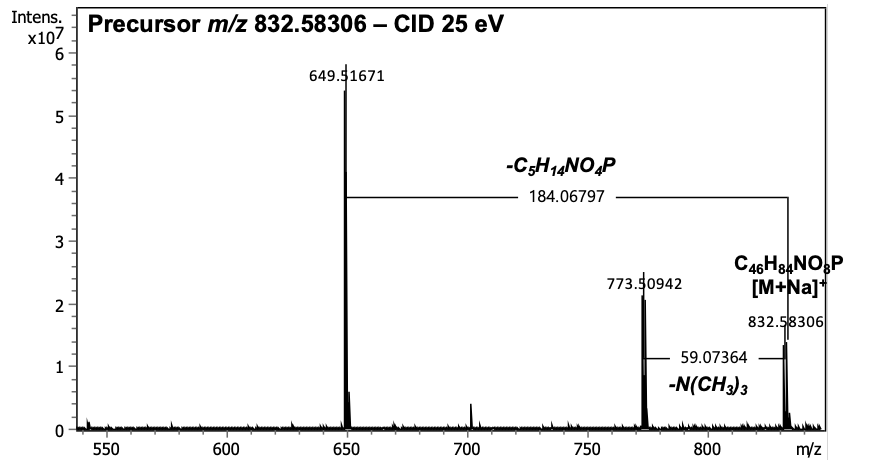
**

**Supplementary Figure 9**. MALDI-MS/MS spectra obtained from phosphatidylcholine 38:4 (PC 38:4, precursor *m/z* 832.58306, [M+Na]^+^) analyzed in positive ion mode by using DHB as a matrix Spectra obtained using a Bruker solariX MALDI-FTICR MS 12 Tesla. Collision induced dissociation (CID) at 25 eV was used for acquiring the MS/MS spectra.

**
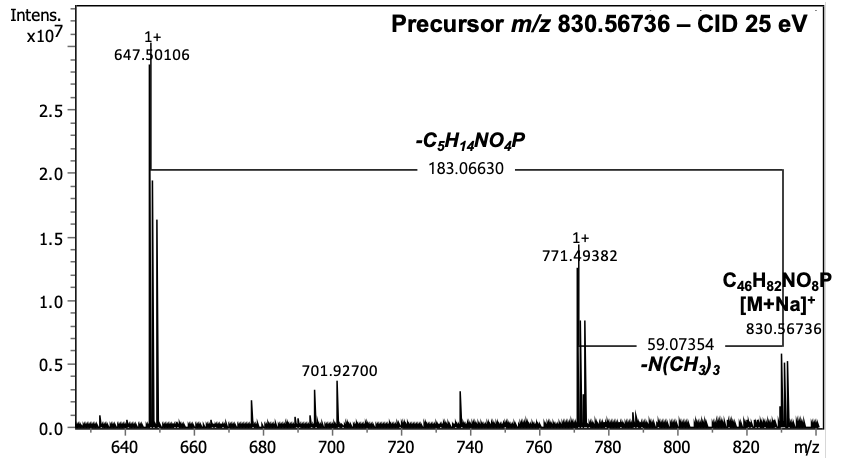
**

**Supplementary Figure 10**. MALDI-MS/MS spectra obtained from phosphatidylcholine 38:4 (PC 38:4, precursor *m/z* 832.58306, [M+Na]^+^) analyzed in positive ion mode by using DHB as a matrix Spectra obtained using a Bruker solariX MALDI-FTICR MS 12 Tesla. Collision induced dissociation (CID) at 25 eV was used for acquiring the MS/MS spectra.


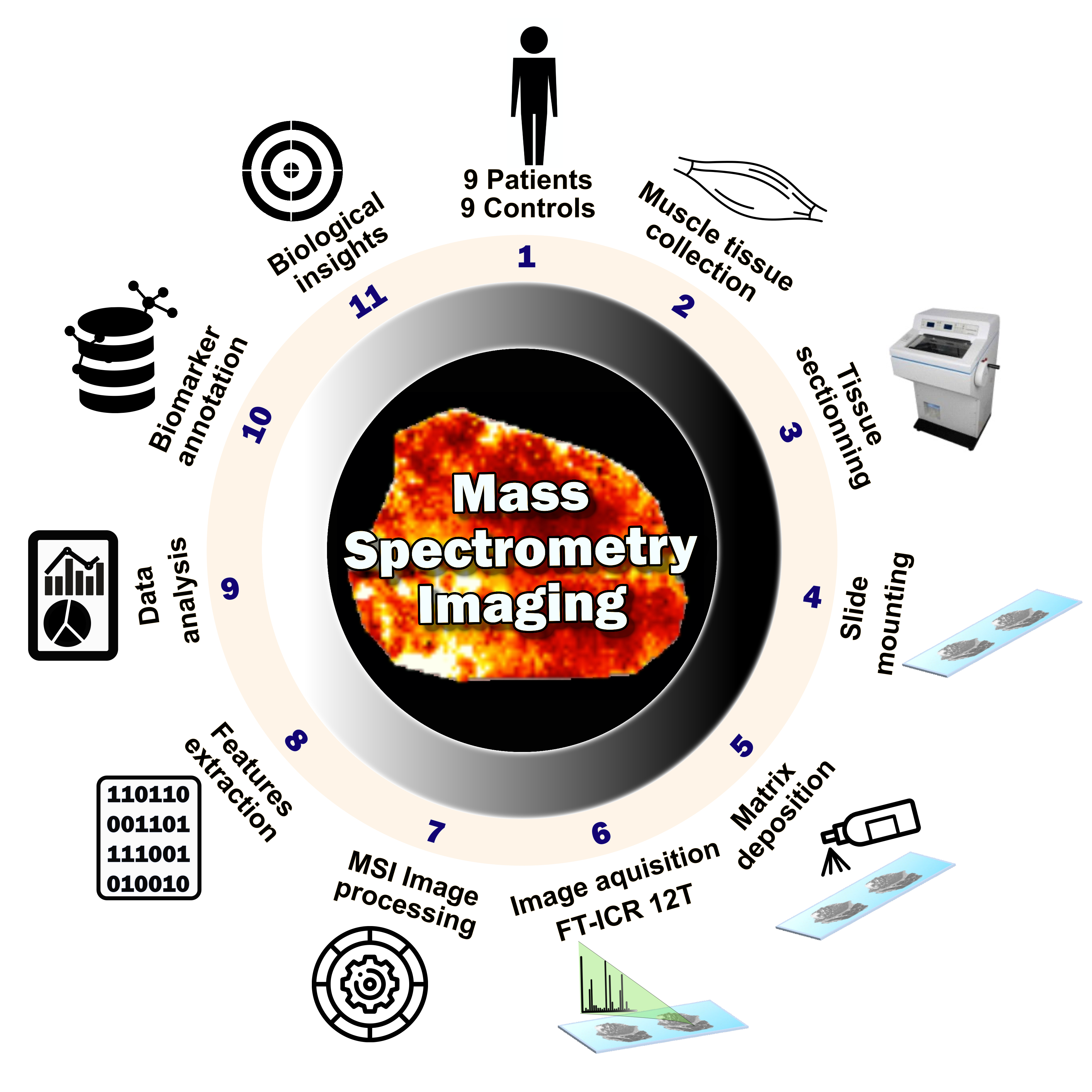


**Supplementary Figure 11**. **General overview of mass spectrometry imaging workflow**. (1) Patient selection; (2) Tissue sampling; (3) Tissue sectioning using a cryomicrotome; (4) Tissue section fixed to the sample probe; (5) Homogenous deposition of matrices using an automatic sprayer; (6) MSI data acquisition using an FT-ICR 12T Solarix Bruker; (7) Mass spectrometry imaging datasets (raw data files) were processed; (8) Raw data were converted into data matrices, including ion mass-to-charge ratios (m/z), spatial localization and relative intensity for each ion image, were generated using SCiLs Lab software; (9) Data analysis workflow for comparative analysis to extract the differential metabolic patterns; (10) Annotation of the discriminant features by interrogation mass spectrometry databases; (11) Interpretation of the data according to clinical and biological contexts.
